# Endoscopic recanalization for the complete closure of long-gap esophageal atresia after reconstruction surgery

**DOI:** 10.1186/s12876-022-02207-y

**Published:** 2022-03-22

**Authors:** Shin Kashima, Kentaro Moriichi, Yu Kobayashi, Yuya Sugiyama, Yuki Murakami, Takahiro Sasaki, Keitaro Takahashi, Katsuyoshi Ando, Nobuhiro Ueno, Hiroki Tanabe, Mikihiro Fujiya

**Affiliations:** Division of Metabolism and Biosystemic Science, Gastroenterology, and Hematology/Oncology, Department of Medicine, Asahikawa Medical University, 2-1 Midorigaoka-higashi, Asahikawa, Hokkaido 078-8510 Japan

**Keywords:** Long-gap esophageal atresia, Complete esophageal closure, Puncture needle, Endoscopic recanalization

## Abstract

**Background:**

Reconstruction surgery-associated stricture frequently occurs in patients with long-gap esophageal atresia (LGEA). While several endoscopic dilatation methods have been applied and would be desirable, endoscopic recanalization is very difficult in cases with complete esophageal closure. Surgical treatment has been performed for a severe stricture, which causes extensive damage to the infant. No reports have described successful endoscopic recanalization for complete closure due to scarring after surgery for LGEA. We herein report the case of successful endoscopic recanalization by single endoscopist in an LGEA patient with complete closure after reconstruction surgery.

**Case presentation:**

A seven-month-old boy with LGEA who received reconstruction surgery and gastrostomy immediately after birth presented to our unit due to vomiting and malnutrition. Contrast radiography and peroral endoscopy detected complete closure of the esophagus at the anastomotic site. After confirming the length of stricture as several millimeters, we punctured the center of the lumen with a 25-G puncture needle under fluoroscopy. An endoscope was then inserted via the gastrostomy and the puncture hole was detected at the center of the lumen. After passing the guidewire, endoscopic balloon dilation was performed three times, and the hole was sufficiently dilatated. Oral ingestion was feasible, and his nutritional condition was improved.

**Conclusions:**

To our knowledge, this is the first report to propose a less invasive endoscopic approach to recanalize a site of complete esophageal closure in a LGEA patient after reconstruction surgery by single endoscopist. Our endoscopic procedure using an ultrathin endoscope and puncture needle may be a therapeutic option for the treatment of patients with complete esophageal closure in a LGEA patient after reconstruction surgery.

## Background

Esophageal anastomotic stricture remains one of the main complications after the surgical repair of esophageal atresia [1]. Esophageal stricture (ES) is reported to be caused by the suture technique, anastomotic leakage and tension, long gap esophageal atresia (LGEA) and gastro-esophageal reflux disease [2]. In most cases ES is treated by endoscopic balloon dilation, which is well accepted worldwide, because of its efficacy and lower degree of invasiveness [3]. However, in the case of a severe stricture, such as pinhole or complete esophageal closure at the anastomotic site, which the guidewire cannot pass through, surgical treatment, such as stricture resection or esophageal substitution, has generally been performed. This approach causes extensive damage to the infant. No reports have described successful endoscopic recanalization for complete esophageal closure due to scarring after surgery for LGEA.

This report describes a less invasive endoscopic approach by single endoscopist that was successfully applied in the recanalization of complete esophageal closure at the anastomotic site in an LGEA patient who had undergone reconstruction surgery.

## Case presentation

**Fig. 1:**
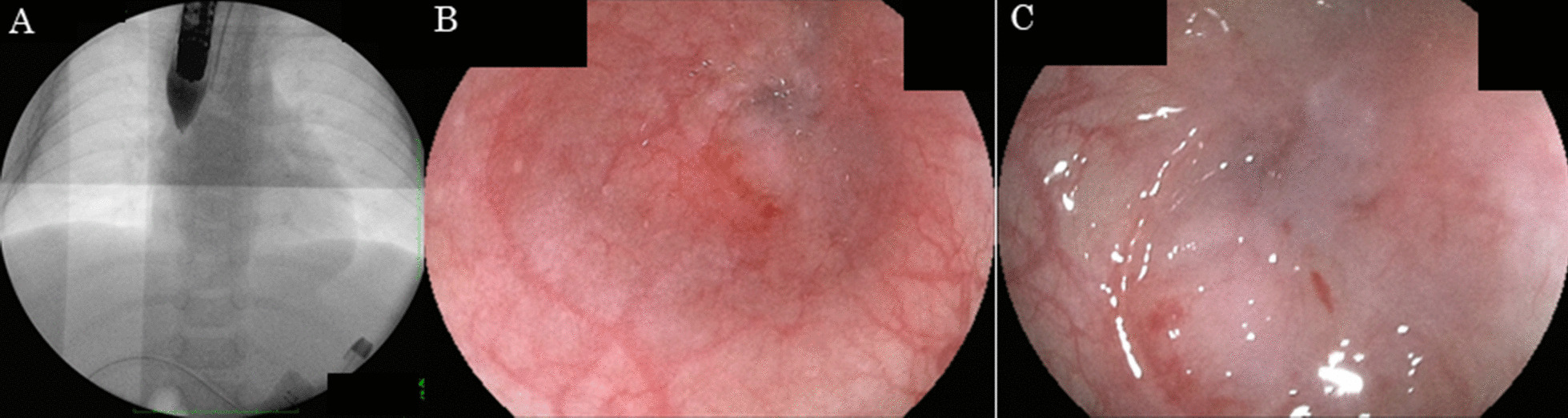
Contrast radiography (**A**) and the endoscopic findings (peroral) at a distant view (**B**) and close-up view (**C**) detected the complete closure of the esophagus at the site of anastomosis

**Fig. 2:**
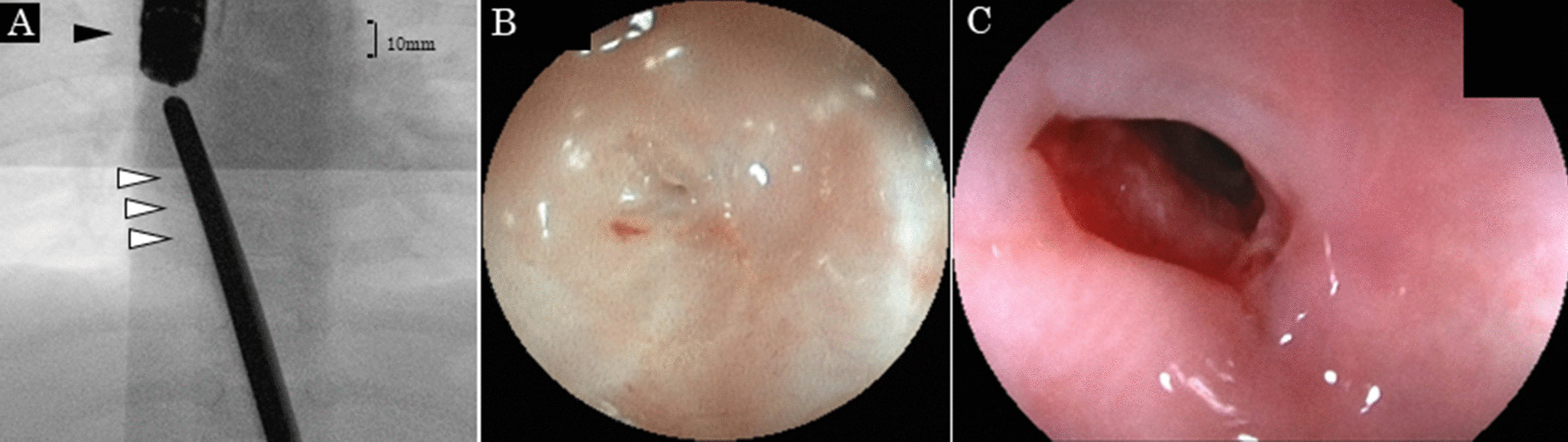
Fluoroscopic and endoscopic findings after puncture and endoscopic balloon dilation. **A** Pean forceps were inserted via the gastrostomy toward the caudal side of the anastomotic site. Contrast radiography showed that the length of the stenosis was several millimeters (black arrow, peroral conventional endoscope; white arrow, Pean forceps via gastrostomy). **B** An ultrathin endoscope was inserted via the gastrostomy after the puncture. The puncture hole was present at the center of the lumen. **C** After endoscopic balloon dilatation had been performed three times, the hole was deemed sufficiently dilated

A seven-month-old boy presented to our unit due to vomiting and malnutrition. He had been diagnosed with esophageal atresia Gross type A and received gastrostomy immediately after birth. At one month of age, esophageal extension with Howard’s method had been performed due to a long gap. At four months of age, reconstruction surgery was performed after sufficient esophageal extension was confirmed. However, immediately after reconstruction surgery, minor suture leakage occurred, and severe stenosis was observed. It was thought that the leakage had caused the severe stenosis at the anastomotic part. Subsequently, balloon dilation under fluoroscopy without endoscopy was performed by pediatric surgeons twice to prevent the progression of severe esophageal stricture. The pediatric surgeons decided not to leave the nasogastric tube in place, as the fluoroscopic examination revealed that the stenosis after balloon dilation was not severe. Even though the stenosis was successfully dilated, the infant had to be fed most supplementary enteral nutrition via gastrostomy because of aspiration due to a poor swallowing function when he was discharged from the hospital. Unfortunately, the patient was unable to undergo a medical examination after the second balloon dilation procedure due to family circumstances. At the present (second) admission, pediatric surgeons tried to perform balloon dilation again under fluoroscopy without endoscopy; however, the stenosis lesion was not outlined. They abandoned balloon dilatation without endoscopy. Our department was thus asked to perform balloon dilation with endoscopy under direct observation. Contrast radiography and peroral endoscopy detected complete esophageal closure with scarring at the anastomotic site because of the discontinuation of balloon dilation (Fig. 1). However, we suspected that this stenosis was not "refractory stricture” and was indicated for balloon dilation, as only two balloon dilations had been performed before. After confirming the direction of the lumen at the caudal end and the length of stenosis as several millimeters (Fig. 2A), we punctured the center of the lumen with thin puncture needle (needle thickness; 25 G, needle length; 4 mm, outer diameter of the tube; 1.75 mm, Sumitomo Bakelite Co., Ltd., Tokyo, Japan) toward the marking under fluoroscopy. After completing the puncture, the peroral endoscope was withdrawn and an ultrathin endoscope was inserted via the gastrostomy in order to confirm the created hole. The puncture hole at the center of the lumen (Fig. 2B) was confirmed and the hole was subsequently re-punctured from the distal side through the ultrathin endoscope, then the guidewire was successfully passed through the stricture. After passing the guidewire, the ultrathin endoscope was withdrawn, and under peroral endoscopy, endoscopic balloon dilation was performed safely using the guidewire. Eventually, we performed endoscopic balloon dilation three times (once a week for three weeks), and then the hole was sufficiently dilatated and successfully observed epithelization of the lesion (Fig. 2C).

After endoscopic balloon dilations, to secure patency, a nasogastric tube was retained, and balloon dilation by pediatric surgeons without endoscopy was performed five times (once a month for five months) based on the severity of the stenosis evaluated by fluoroscopy. We eventually confirmed stabilization of post-recanalization esophageal stenosis via a fluoroscopic examination after five balloon dilations. Consequently, oral ingestion was feasible, and his nutritional condition improved. Three years have passed since then, and despite balloon dilation not being performed, sufficient oral intake is now possible with a small amount of supplementary enteral nutrition.

## Discussion and conclusions

We herein report the first case of successful endoscopic recanalization by single endoscopist in an LGEA patient with complete esophageal closure with scarring after reconstruction surgery.

Reconstruction surgery-associated stenosis frequently occurs in LGEA patients [1]. Several endoscopic dilatation methods have been applied and their efficacy has been reported [3]. However, surgical treatment, such as stricture resection or esophageal substitution, has been performed in cases of a severe stricture because it is difficult to achieve recanalization by endoscopic dilation in such cases. While surgical treatment is effective for treating severe stenosis, it is much more invasive than endoscopic treatment, and causes extensive damage to the infant. While endoscopic treatment would be desirable, endoscopic recanalization is very difficult in cases with complete esophageal closure. In addition, endoscopists had to ensure the patient was indicated for the procedure. This patient had undergone balloon dilation twice, so the stenosis was not considered "refractory stricture” and was indeed indicated for balloon dilation. When performing recanalization in cases with complete esophageal closure, it is extremely important to secure the safety of the procedure. To pass athe guidewire, it is crucial to estimate the puncture line. We decided the puncture line while changing the position under fluoroscopy. Takamizawa et al. reported that a severe stricture was successfully treated with magnetic compression [4]. Their method required passage of a guidewire through the esophageal stricture and several expansions of the gastrostomy site were needed to pass the magnet. Pane et al. reported the successful recanalization of a severe stricture using two endoscopes simultaneously [5]. Two endoscopes were inserted (per oral and through gastrostomy) and the stenotic site was then punctured with a guide with trans-illumination from the endoscope on the opposite side. However, this technique requires multiple endoscopic light source devices and multiple endoscopists. In addition, it seemed difficult to utilize trans-illumination for the length of the stricture and/or in cases involving flexure. Unlike the abovementioned report, our procedure could easily recanalize a site of complete esophageal closure using one endoscopic light source and a single endoscopist. When puncturing, we chose a thin puncture needle because we judged—based on the examination before the puncture—that the length of the puncture needle was long enough to penetrate the site of complete esophageal closure through even the ultrathin endoscope. Additionally, several expansions of the gastrostomy site were unnecessary. Thus, using our procedure, a site of complete esophageal closure with scarring could be easily and rapidly recanalized. This approach was less invasive to the infant in comparison to previously reported procedures.

To prevent recurrence of esophageal complete stricture, retainment of a nasogastric tube and balloon dilation may be essential, and repeated dilations should be performed until the confirmation of stabilization of post-recanalization esophageal stenosis by endoscopy or fluoroscopy. Our procedure was associated with some limitations. First, the length of recanalization that can be achieved depends on the length of the needle. Needles of various lengths are commercially available. To the best of our knowledge, the maximum needle length is eight millimeters. Puncture would be difficult in cases in which the length of stricture exceeds eight millimeters. In cases in which the length of the complete stricture exceeds eight millimeters, endoscopic procedures for recanalization, including those described in previous reports, should be avoided. If balloon dilations have been performed many times or if various attempts at endoscopic recanalization fail, surgical treatment should be considered, as minor esophageal suture leakage is not thought to cause severe adhesion (frozen mediastinum). Second, the safety of the procedure should be elucidated. To secure the safety, we carefully set a marker after confirmation of the direction for puncture under fluoroscopy, and then performed the puncture. However, at the present time, there is no established safe procedure for patients with complete esophageal closure. A further analysis with a larger study population is needed to reveal the efficacy and safety of our approach.

In conclusion, this report described a less invasive endoscopic approach for the recanalization of a site of complete esophageal closure that was successfully applied in the treatment of an LGEA patient after reconstruction surgery. Although there are still several problems, including the length of stenosis and safety, our endoscopic procedure allowed recanalization to be easily performed by single endoscopist and one endoscopic light source. This approach may be a therapeutic option for the treatment of patients with complete esophageal closure.

## Data Availability

The datasets used and/or analyzed during the current study available from the corresponding author on reasonable request.

## Abbreviations

LGEA: Long-gap esophageal atresia
ES: Esophageal stricture
